# The CSL proteins, versatile transcription factors and context dependent corepressors of the notch signaling pathway

**DOI:** 10.1186/s13008-016-0025-2

**Published:** 2016-09-27

**Authors:** Humberto Contreras-Cornejo, Germán Saucedo-Correa, Javier Oviedo-Boyso, Juan José Valdez-Alarcón, Víctor Manuel Baizabal-Aguirre, Marcos Cajero-Juárez, Alejandro Bravo-Patiño

**Affiliations:** 1 Centro Multidisciplinario de Estudios en Biotecnología (CMEB) de la Facultad de Medicina Veterinaria y Zootecnia, Universidad Michoacana de San Nicolás de Hidalgo, Posta Veterinaria, Km. 9.5 Carretera Morelia-Zinapécuaro, Col. La Palma, C. P. 58890 Tarímbaro, Mich. México; 2 Instituto de Investigaciones Agropecuarias y Forestales (IIAF), Universidad Michoacana de San Nicolás de Hidalgo, Km. 9.5 Carretera Morelia-Zinapécuaro, Col. La Palma, C. P. 58890 Tarímbaro, Mich. México

**Keywords:** Notch signaling pathway, CSL, Hairless, Negative regulation, Embryo development

## Abstract

The Notch signaling pathway is a reiteratively used cell to cell communication pathway that triggers pleiotropic effects. The correct regulation of the pathway permits the efficient regulation of genes involved in cell fate decision throughout development. This activity relies notably on the CSL proteins, (an acronym for *C*BF-1/RBPJ-κ in *Homo sapiens*/*Mus musculus* respectively, *S*uppressor of Hairless in *Drosophila melanogaster*, *L*ag-1 in *Caenorhabditis elegans*) which is the unique transcription factor and DNA binding protein involved in this pathway. The CSL proteins have the capacity to recruit activation or repression complexes according to the cellular context. The aim of this review is to describe the different co-repressor proteins that interact directly with CSL proteins to form repression complexes thereby regulating the Notch signaling pathway in animal cells to give insights into the paralogous evolution of these co-repressors in higher eumetazoans and their subsequent effects at developmental processes.

## Background

In the different species where the Notch signaling pathway (NSP) has been described, the activator complex seems to be widely conserved in its structure and function, whereas the repression complex is surprisingly diverse. This review aims to identify and describe different co-repressor proteins that interact directly with CSL proteins to form nuclear repression complexes within specific cells, tissues and different states of development that modulate in a negative fashion Notch dependent gene transcription. Hence we try to deepen on different proteins which share the capability of assembling complexes with the CSL protein family, as well as: CBF-1 interacting repression (CIR) protein, SPEN-SHARP/MINT protein family, Insensitive and BEND6 proteins, KyoT 2 and KyoT 3 proteins, and RITA protein. All of them are specialized in antagonizing gene expression engaging NSP at different cellular contexts.

## Introduction

In a multicellular organism, cell fate is specified by a complex interplay between signaling pathways during embryo development. This cross talk is fundamental for cell differentiation, cell proliferation, cell migration and patterning of highly organized tissues [1–3]. Development of a three-dimensional, fully functional biological structure requires cellular assembly to be extremely precise and depends on the cellular context. Cells use these mechanisms to sense their environmental conditions with the purpose of taking a final decision.

In this context the NSP is essential to allow a highly regulated cross talk between cells to coordinate their complex organization in space and time during embryo development. It should be clear that the NSP ends in a selective modulation of the expression of target genes that themselves encode transcriptional regulators. As a consequence, these transcriptional regulators have the capacity to affect the expression of other genes that modify cellular activities such as cellular differentiation, stem cells maintenance, apoptosis and other pleiotropic effects [1, 4–7]. This regulatory mechanism is important during metazoan embryo development and is highly conserved among different animal models where the pathway has been described [8–11].

**Fig. 1:**
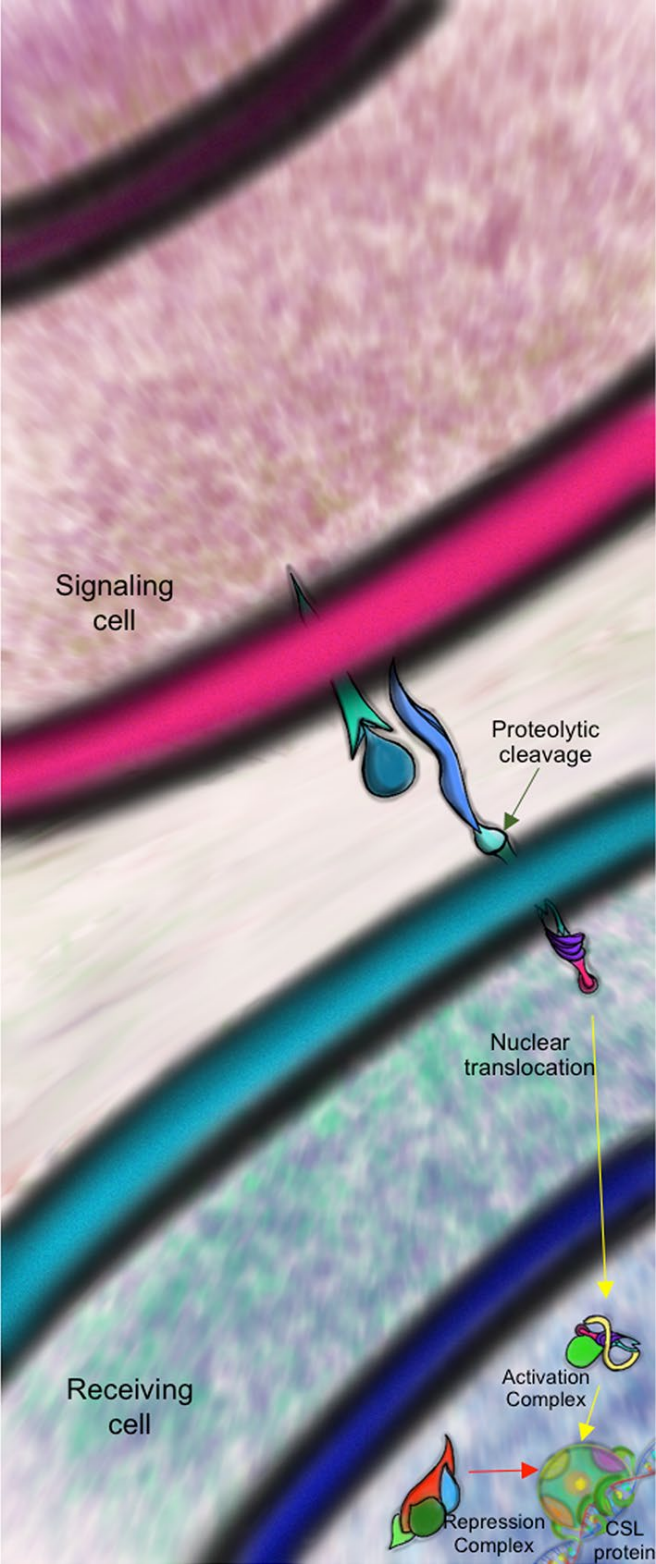
General view of the NSP: once a ligand of the DSL family in the signal sending cell interacts with the Notch receptor of the signal receiving cell, NICD is released by proteolysis and translocated to the nucleus activating the pathway for a positive gene regulation together with CSL proteins, Mastermind (Mam) protein and histone acetyltransferases (HATs). Otherwise NSP is repressed by “default repression” complexes structured by Hairless (H) protein and C-terminal binding protein (CtBP) and Groucho (Gro) co-repressors

**Fig. 2:**
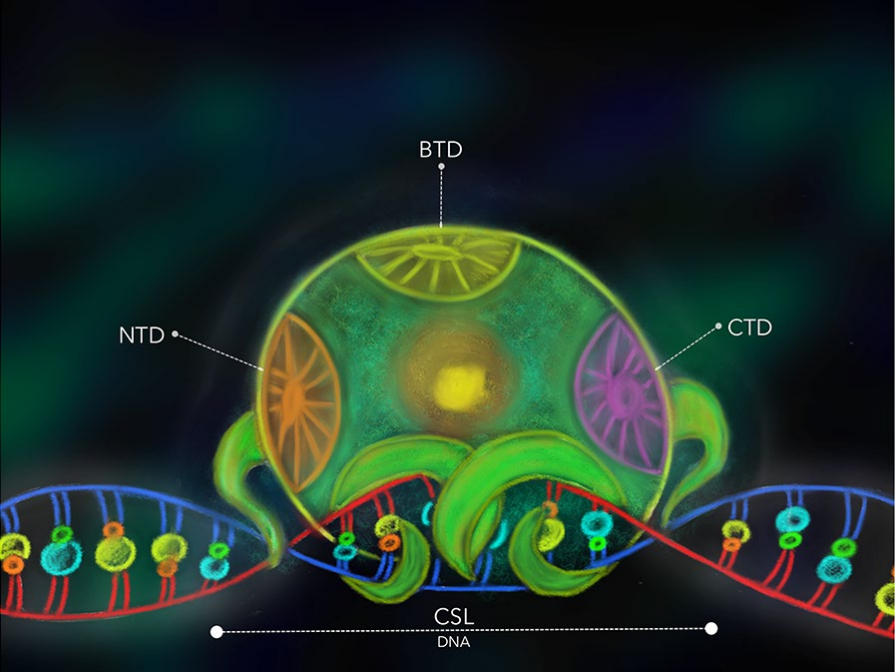
The CSL proteins: CSL proteins are transcription factors regulating the Notch pathway in a positive and negative fashion. CSL type transcription factors have three functional domains well characterized: N-terminal domain (NTD), beta-trefoil domain (BTD) and C-terminal domain (CTD) which are used for protein–protein or protein-DNA interactions

Briefly NSP works as follows (Fig. 1): once a protein of the DSL family (*D*elta/*S*errate in *Drosophila melanogaster*; *L*AG-2 in *Caenorhabditis elegans*; Dll-1-4/Jagged in mammals) in the signal sending cell contacts the receptor, a protein of the Notch family (Notch, *D. melanogaster* and mammals; Lin-12/Glp-1, *C. elegans*) in the signal receiving cell, the NSP is activated by proteolysis of the Notch receptor. This proteolysis process releases the Notch intracellular domain (NICD) from the cell membrane [7, 12, 13]. NICD is translocated to the cell nucleus where, together with CSL proteins (Fig. 2), a fully functional transcription activation complex is assembled, including other co-activator factors such as Mastermind protein (Mam, *D. melanogaster*; LAG-3, *C. elegans*; MAML, mammals) [13–15], SKIP [16] and histone acetyltransferases (HATs) [15, 17]. This complex modifies the chromatin and activates gene expression at specific loci depending on the precise cellular context [7, 18–21].

**Fig. 3:**
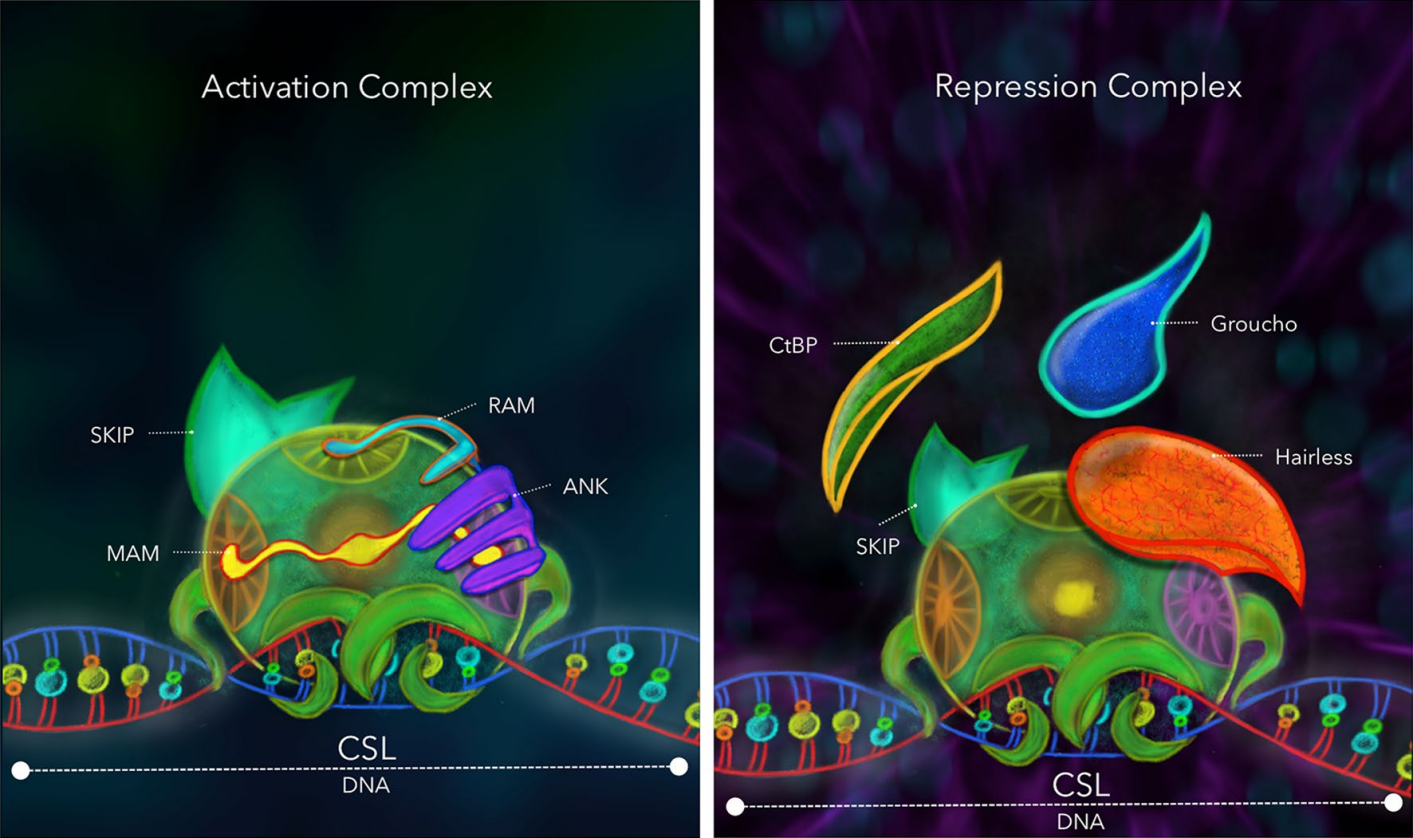
Comparative view of the repression and activation complexes. One of the best known models of the NSP is *Drosophila melanogaster*. CSL transcription factor acts as a bridging protein between the DNA and a complex of proteins intended to modify chromatin topology in a specific locus. In the case of the gene repression complex, CSL recruits H that in turn will form a HDAC together with Gro and CtBP. Even if H is the main co-repressor of the pathway in the fly fruit, no H homolog has been found in models out of insects, but instead a series of other proteins seem to take this function as we will see further. For the gene activation complex, NICD and Mam occupied the CSL’s domains and, in turns, recruit a HAT complex to generate an open chromatin topology and promote gene expression. Activation complexes seem to be similar in all models where NSP has been studied. Skip is common in both complexes

While the activator complex seems to be widely conserved in its structure and function, the repression complex is surprisingly diverse in the different species where the NSP has been described [17]. But what exactly is the role of the repression complex? In the absence of NICD the CSL transcription factor functions as a transcriptional repressor in a “default repression” fashion. In this case CSL recruits co-repressor proteins such as Hairless (H) [22], *C*-*t*erminal *b*inding *p*rotein (CtBP), Groucho (Gro) [23, 24] and Insensitive [25] in *D. melanogaster* or SMRT/NCoR, CIR, KyoT proteins, SHARP/MINT in mammals [26, 27] as well as Sin3A and KDM5a and histone deacetylases (HDAC) [6, 9, 18, 28, 29]. This activity switch depends on the precise cellular context during the regulatory process which implies conformational chromatin variations caused by quick exchange between factors required to activate or repress transcription [3, 5, 18, 30] (Fig. 3).

In the fruit fly, NICD activity is antagonized by H protein [17, 22, 31–33] (Fig. 3), a process critical and indispensable for correct tissue development, especially during cell differentiation events. Numerous genetic studies in the past have revealed that the gene dosage of NSP members is critical for fly development, and that Notch (N) and H proteins conserve a 1:1 ratio to assure the correct NSP function. For example single mutations of both N (*N* −/+) or H (*H* −/+) show a dominant loss of function phenotype in the adult wings and bristles (mechanosensory organs of the peripheral nervous system). However, in double mutants (*N* −/+; *H* −/+) the wings are almost wild type [22, 32, 34, 35]. In other words, H functions in a dose dependent manner, reflecting the strategic role of this protein in the NSP [22], with the peculiarity that, until now, H protein has been identified only in insects [33, 36]. It has been shown that the H protein directly interacts with the CSL protein Su(H), and assembles a repressor complex by recruiting Gro and CtBP co-repressors [18, 22, 23]. The resulting repressor complex leads to the repression of the NSP target genes [36]. This means that the activity of the activation complex (NICD/Su[H]) or the repression complex (H/Su[H]) allows differential genes expression of the bHLH family, driving to a specific cell destination, depending on the precise cellular context, during differentiation processes at early development states and beyond.

As shown in Fig. 2, CSL proteins consist of three domains: N-terminal domain (NTD), beta-trefoil domain (BTD) and C-terminal domain (CTD) [37, 38]. The NTD and BTD make contact with the DNA [38] allowing the recognition of different regulatory sequences on the NSP target genes [39]. When the activator complex is formed, NICD uses the three domains: BTD makes contact with RAM (RBPJ associated molecule) whereas CTD and NTD make contact with the ANK (ankyrin) domains of the activated Notch receptor NICD respectively [40, 41], while the Mam protein lies in a grove contacting the ANK domain of NICD as well as CTD and NTD of CSL [15, 42, 43]. When the repression complex is formed, interactions of the corepressor proteins with CSL differ within the various model organisms. In the case of *D. melanogaster* H protein interacts with the CTD domain of CSL (Su[H]) [17], but in the case of mammals the relevant sites for interaction with corepressor proteins lie within the BTD domain as we will see below. Hence, in mammals a competition of corepressors and NICD for CSL protein binding is very likely [17].

NSP activity has been reported beyond embryo development to guarantee the correct cell and physiological function in mammals. The incorrect signaling is turned into health disorders, such as cancer [44–47]. NSP regulates post-embryonic cell specification in invertebrates as well. In *D. melanogaster* for instance, through the *Achaete*-*Scute* complex, a single cell is selected within a so-called proneural cluster to become a sensory organ precursor cell (SOP) [48]. This cell will activate the Notch receptor in the neighboring cells so they will take an epidermal fate through the differential expression of the bHLH transcription factors of the *Enhancer of split*-complex [E(spl)-C]. In subsequent divisions the SOP gives rise to five different cells forming a mechano-sensory organ. Inhibition of the proneural fate and cell fate specification, respectively, is carried out by the *E(spl)* family genes [1, 2] and other NSP regulators such as H [48], Insensitive [25], Insensible [49] and Numb [1]. These are examples of factors that, in specific cell contexts exert negative regulation of the NPS. Some of these proteins have the capacity to form complexes with CLS and regulate in a negative way the expression of NSP target genes [7, 9, 50, 51].

After fertilization, the first cell differentiation events in vertebrate embryogenesis occur at the transition from morula to blastocyst stage. Two cells types arise: embryoblasts, which are constituted by the internal cell mass (ICM); and trophoblasts forming a cell layer also called trophectoderm epithelium (TE) surrounding the blastocoel [4]. The second round of cell differentiation events occurs after cell proliferation and space rearrangements of blastomeric cells, during the transition from blastocyst to gastrula. Two superposed cell layers appear: epiblast and hypoblast. Epiblast gives rise to embryonic epiblast and amniotic ectoderm; whereas hypoblast originates extra embryonic endoderm and the embryonic sac. Simultaneously, cellular specialization of the embryonic epiblast gives rise to two cell layers: ectoderm and primitive streak. The primitive streak eventually forms mesoderm and endoderm. Up to now, the embryo has completed the three germ cell layers (ectoderm, mesoderm and endoderm), precursors of different highly specialized cell types [4].

The nervous system is the first defined tissue in the developing embryo. It derives from the ectoderm through the neural groove rearrangement, followed by mesodermal cell compaction that gives rise to somites, which are aligned along the antero-posterior axis of neural notochord. This is the beginning of the cell specialization processes, where NSP is fundamental to obtain specialized tissue from ectoderm, mesoderm and endoderm [4].

It has been observed that in metazoan embryos, a generating zone of growing cells moves into the anterior site of presomitic mesoderm (PSM). Here, segmentation occurs by compaction and adhesion of cells from the PSM, followed by epithelium coating and separation of single structures. This rearrangement generates somite pairs in an antero-posterior orientation, in a specific number and time for each vertebrate species [4, 52, 53]. All these processes are strictly regulated in time and space during embryonic morphogenesis. To explain these phenomena, a “Clock and wavefront model” has been proposed [54, 55] and extensively reviewed by Özbudak and Pourquié [56]. This model proposes the existence of a clock or of systematic biochemical oscillations within the PSM cells. The whole process coordinates a cell response to the big wave of signals that comes from the anterior segment of the PSM. Three signaling pathways are described as responsible for the biochemical oscillation within the PSM cells: NSP, FGF and Wnt. In this context, it is essential to clarify that although each signaling pathway seems to stabilize its own oscillation in an independent way from the other two, NSP acts as a “coupling device”. This “device” synchronizes all the process that will give rise to highly specialized cellular lineages at the physiological level [56, 57].

What is the molecular basis for the “coupling device”? Axin2 kinase regulates the stability of beta-catenin, the transcriptional co-activator in the Wnt signaling pathway. Wnt is capable of regulating the *Lunatic fringe* (*Lfng*) gene expression in a negative way through the Gsk3/beta-catenin activity [56, 57]. Lfng is a glycosyltransferase, regulating Notch substrate sensitivity by direct glycosylation of the receptor, and inhibiting Notch signaling in this context. Activation of the Notch receptor at the cell membrane results in the release of NICD. If NICD is released and reaches the cell nucleus, it interacts with CSL proteins. This NICD/CSL complex exerts a positive regulation over the expression of a number of genes, among them, *Lfng* itself. In other words, a reduction of Lfng concentration results in an increase of NICD activity. This means that a negative feedback exists between NICD and Lfng which is potentiated by Axin2 activity [56–58].

FGF activates the *T*-*box* gene family and the *Dusp6* gene. The *T*-*box* gene family encodes Tbx transcription factor proteins that are involved in the specification of paraxial mesoderm structures and also play an essential role in left/right axis determination [4]. *Dusp6* gene encodes Dusp6 protein, a member of the dual specificity protein phosphatase subfamily. This protein negatively regulates members of the mitogen-activated protein (MAP) kinase superfamily (MAPK/ERK, SAPK/JNK, p38), which are important for cell proliferation and differentiation [56, 57]. Tbx6 protein exerts a positive regulation on *Delta*-*1* gene expression, inducing a suitable expression of Delta-1 ligand. This ligand allows cells located at the anterior and posterior regions of a somite to induce the NSP resulting in the expression of some members of the basic helix-loop-helix (bHLH) protein family (transcription factors). Those “activated” cells will be allowed to follow different cellular lineages. At this point, Tbx24 protein becomes essential to determine the limits of each somite and its final maturation [53, 56–58].

The own nature of a clock or a biochemical oscillation implies that every signal wave should return to the equilibrium point from where it began [4]. In the case of the “Clock and wavefront model” or segmentation clock, the activation period of the NSP during the successive rounds of the formation of somites happens by the mechanism of negative transcriptional feedback, which works as follows: the NICD/CSL complex induces the expression of transcription regulators necessary for *Delta*-*1* expression which reaches relatively high concentrations [12, 52]. At this point, ubiquitination of NICD and the inhibition of transcriptional regulators cause the inhibition of *Delta*-*1* expression. In mouse, for example, the oscillation of ligand expression happens every 2 h, which agrees with the time of somite formation in this mammal [58].

As previously described, fluctuations on Lfng concentration ultimately result in changes of NICD activity. This cyclic activation/inhibition of Notch receptor also corresponds to oscillations in the Delta-1 concentration levels. This supports a suitable synchronization of all events of specialization that occur in somite formation, where NSP activity is essential to regulate segmentation of the embryo [3, 58, 59].

In the past, a vast array of information has been collected, describing molecular events on the processing of NSP components to achieve an accurate pathway’s function: location of ligand and receptor at cell membrane [60–64]; differential interaction between ligands and receptors mediated by site specific glycosylation [7, 20, 59, 65–70]; protein stability, exchange rate and transcriptional complex destabilization mediated by protein phosphorylation [71, 72] or methylation [73]; receptor activation and protein exchange rate mediated by proteolysis [3, 74, 75].

Moreover, numerous details on the activation/inactivation of the transcriptional regulators exerting NSP function during cell differentiation processes have been described. CSL proteins mediate this important task because, as transcriptional factors, they have the capacity to recognize the consensus DNA sequences found in the genes regulated by NSP [39]. In absence of NICD, the CSL transcription factor works as a repressor “by default”, suppressing spurious genes expression [19, 50]. Notch receptor activation results in NICD release, which causes replacement of the transcriptional repression complexes by activation complexes, allowing the gene expression [13, 76, 77]. This exchange of transcriptional regulatory complexes supports, in part, the correct “fine-tuning on/off” depending on the cellular context where NSP is working [9, 17, 78–82]. This review aims to identify and describe different co-repressor proteins that interact directly with CSL proteins to form nuclear repression complexes within specific cells, during embryo development as well as later on, which modulate in a negative fashion Notch dependent gene transcription.

### CBF-1 interacting repression (CIR) protein

**Fig. 4:**
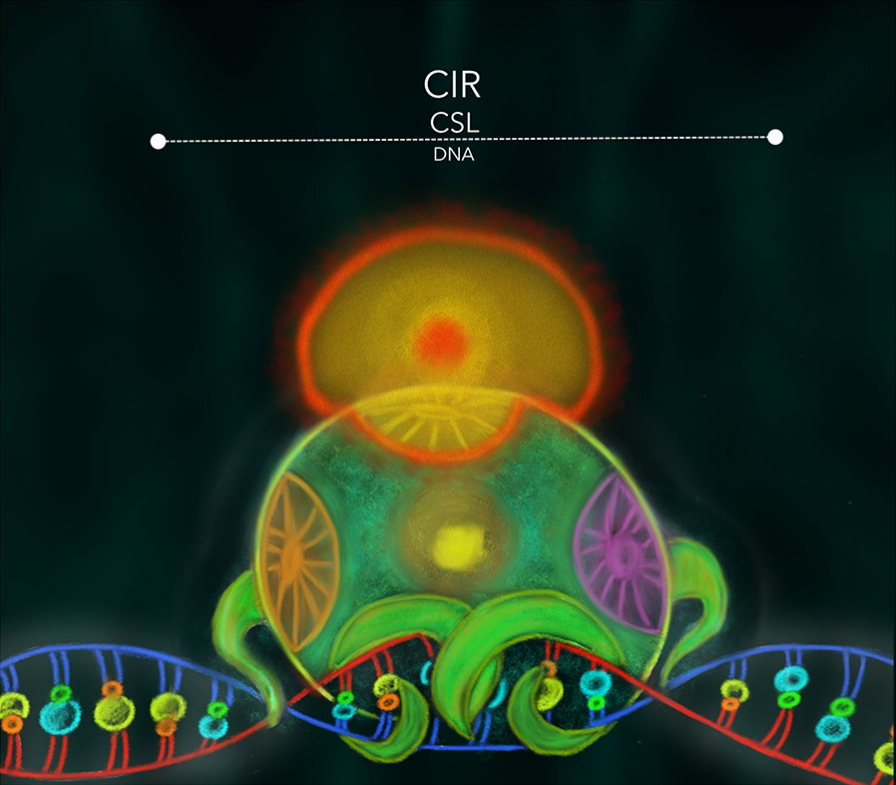
CIR-CSL interaction: CBF-1 interacting region for CIR is located at the BTD of CBF-1 (a CSL protein) that matches the domain used by the RAM domain of NICD. This interaction suggests a competition for the transcription factor, regulating in a negative fashion genes regulated by NSP in a specific cell context

Hsieh et al. (1999) [83] first isolated the CBF1 interacting corepressor CIR (for *C*BF1 *i*nteracting *r*epressor) out of human B cells. This protein is evolutionarily conserved from man to worm as a homologue was identified in the *C. elegans* sequence. A highly conserved region located between amino acids 1 and 240 contains a CBF-1 (CSL) interaction domain. The CBF-1 interaction region for CIR was mapped between amino acids 233 and 249 [83], at the beta-trefoil domain (BTD) of CBF-1 (Fig. 4). The CIR binding site is conserved in all CSL proteins [17], demonstrating the tight collaboration between CBF-1 and CIR proteins in order to accurately regulate transcription whenever NSP is involved [83].

The *C. elegans* CIR-1 protein homologue [83] is required maternally for early and zygotically for late embryo development; particularly for vulva formation, stem cells maintenance, germ cell development and oocyte differentiation [84]. In *C. elegans* the activity of CIR-1 protein seems to be essential for both, a correct embryo development and sex determination. Human CIR mRNA, however, has been isolated from several other cell types, such as heart, brain, placenta, lung, liver, skeletal muscle and pancreas [83], where CIR protein seems to participate in the appropriate specialized tissue function.

CIR protein functions as a link between CBF-1 (transcriptional factor) and transcriptional co-repressors such as *n*uclear *co*-*r*epressor (NCoR), and silencing mediator retinoid and thyroid hormone (SMRT) as well as histone deacetylases complexes such as HDAC1 and 2, and mSin3 complex, via the Sin3-associated protein of 30 kDa (SAP30) [83]. Actually, chromatin remodeling plays an important role in NSP regulation, as the transcriptional regulatory complexes recruited on the consensus DNA sequence of NSP target genes include several known chromatin remodeling factors [29, 83].

### The SPEN—SHARP/MINT protein family

The human SHARP (from *S*MRT/*H*DAC-1 *a*ssociated *r*epressor *p*rotein) [26, 27], and the mouse homologue MINT (from *M*sx2 *i*nteracting *n*uclear *t*arget) belong to the SPEN (from *sp*lit *en*ds) family of proteins [85, 86]. These proteins all contain RNA Recognition Motifs RRM, and a so-called SPOC domain (*S*pen *p*aralog and *o*rtholog *C*-terminal) [26, 87]. The SPOC domain permits interactions with universal co-repressors such as SMRT, NCoR and CtIP/CtBP proteins [26, 85, 87]. These proteins have the capacity to cooperate with several different transcription factors and regulators, and thus act as negative regulators in various signaling pathways. For example, Shi et al. [88] described the SHARP protein as a negative regulator of the nuclear hormone receptor because of its capability to interact with different corepressor complexes containing HDAC, SMRT, N-CoR and *nu*clear *r*emodeling *d*eacetylase (NuRD). Complexes modify chromatin conformation at regulatory regions of genes controlled by hormones [88].

Likewise, within the NSP context, Oswald and colleagues demonstrated that SHARP physically interacts with RBPJ-κ (CSL) and that it has the capacity to repress transcription mediated by Notch 1 in a HDAC-dependent fashion [26, 27]. At the same time this complex recruits CtBP and CtIP co-repressors [26] which may suggest an alternative mechanism independent of HDAC as reviewed in [89].

**Fig. 5:**
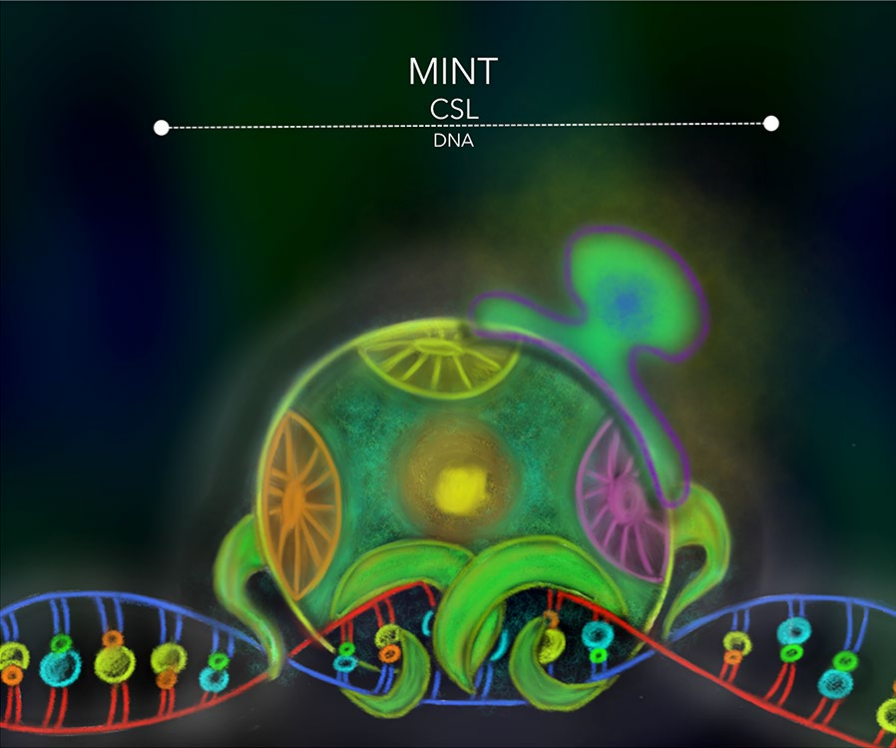
MINT-CSL. The SHARP/MINT proteins were reported to function as negative regulators in diverse cellular contexts as members of a general transcription regulation machinery. MINT is a potent inhibitor of the NSP that uses the BTD and CTD of CSL to form a repression complex with this transcriptional factor to modify the chromatin topology at the NSP dependent genes

MINT protein, originally described in *M. musculus*, presents multiple domains that promote interactions with different transcriptional factors [86, 90]. VanderWielen et al. [82] showed that MINT interacts, via its CID (*C*SL *i*nteracting *d*omain, 2776-2833 aa. (Figure 5), with the BTD and CTD of CSL [82]. In vivo, MINT inhibits NSP during embryo development in the course of cellular specialization during liver, heart, pancreas formation. MINT also regulates the correct differentiation and distribution of lymphocytes. Accordingly, MINT’s inhibition is lethal for mouse embryos [86].

All these data together point to a role of SHARP and MINT as versatile proteins with the capacity to exert negative transcriptional regulation in a highly controlled setting such as in embryo development, where they may be working under the NSP influence, as well as in other cellular contexts as members of the general transcriptional machinery as the literature suggests [26, 82, 85–89]. Notably, despite the similarity of activities between SHARP/MINT and H proteins, there is no homology at the protein level [22, 86]. Moreover, no interaction of SPOC domain containing proteins in *D. melanogaster,* Spen or Spenito, and Su(H) has been described [22, 26]. But a functional homology may be accepted instead.

### Insensitive and BEND6 proteins

The BEN (from *B*ANP, *E*5R and *N*AC1 proteins) domain is present in a great variety of proteins described as negative transcriptional regulators. Typically, these proteins contain one or multiple copies of the BEN domain together with additional characteristic domains [91]. These proteins share several functions: they may act as DNA-binding protein, as chromatin-modifiers or adaptors and have been involved in different signaling pathways [91]. Two of these proteins, referred to as BEN-solo proteins, because they contain a single BEN domain but lacking other motifs, have been related to NSP. The two proteins Insensitive in *D. melanogaster* [25], and BEND6 in mammals (*H. sapiens* and *M. musculus*) [50, 51] are classified as BEN-solo proteins.

The *Insensitive (Insv)* gene in *Drosophila* was identified based on its highly specific expression in sensory organ precursor (SOP) cells [25]. A complete loss of Insv protein has little phenotypic consequences for the fly, unlike a necessary gene for the appropriate development of the peripheral nervous system (PNS). However, elimination of the *Insv* gene generated a gain-of-function of NSP activity in a heterozygous *H* mutant background. In this sensitized background, where NSP activity is already increased due to the limitation of H/Su(H) repressor complex formation, a lack of Insv plays a role: the further increase of NSP activity suggested that Insv normally acts as a corepressor of NSP signaling [25]. Insv is a nuclear protein, which appears to interact via its BEN domain with Su(H) to negatively regulate a reporter gene under the control of the *Enhancer of split*-*C* (*E[spl]*-*C*) promoter [25]. Apparently, Insv can act as a corepressor of Su(H) independent of H. This means, according to Duan et al. [25], that despite Insv protein is expressed within the same cellular context as H protein during *Drosophila* mechano-sensory organ formation, these two proteins probably do not overlap their task during the negative regulation of gene expression [25]. In other words, Insv does not obstruct H protein accumulation and activity; its presence instead may complement H function during *Drosophila* peripheral neurogenesis. Since Insv does not bind directly to the Groucho co-repressor, it seems to mediate repression in a different way as H does [18, 23, 25].

Mammalian BEND6 is likewise capable to interact with CBF-1 protein via its BEN domain [50] and exerts negative transcriptional regulation of NSP target genes, especially during neural differentiation and neural staminal cells (NSC) maintenance [25, 51, 92].

Dai et al. [50] have established some characteristics shared between Insv and BEND6 proteins as antagonists of NSP activity in two different species: (1) both are nuclear proteins exclusively expressed in neural precursor cells and their action is conditioned by the NSP activity during neuron specification. (2) These proteins can interact, in an interspecific way, with the CSL proteins Su(H) and CBF-1 [51]. (3) Both, Insv and BEND6 are capable to repress gene expression regulated by NSP in vivo or in vitro. (4) Because Insv and BEND6 can assemble transcriptional repression complexes involving CSL and NSP proteins, they inhibit bHLH protein expressions to direct a neuronal fate. (5) Insv (fly) and BEND6 (human and mouse) proteins share an identity of 6.7 % at their whole sequence, but share an identity of 33 % at BEN domain. BEND6 proteins from human and mouse share an identity of 84 % [50].

### KyoT 2 and KyoT 3 proteins

The KyoT protein family comprises three isoforms, KyoT 1, 2 and 3, which possess a distinctive LIM-domain-only [93–96]. These isoforms are derived from the same gene via alternative splicing. Isoforms are characterized for containing LIM (from *L*in11, *I*sl-1 and *M*ec-3 proteins) domains [97] arrayed in tandem, with few other sequences outside the LIM domains. The different LIM domains mediate protein–protein interactions [95] thereby regulating transcription processes depending on the cell context.

**Fig. 6:**
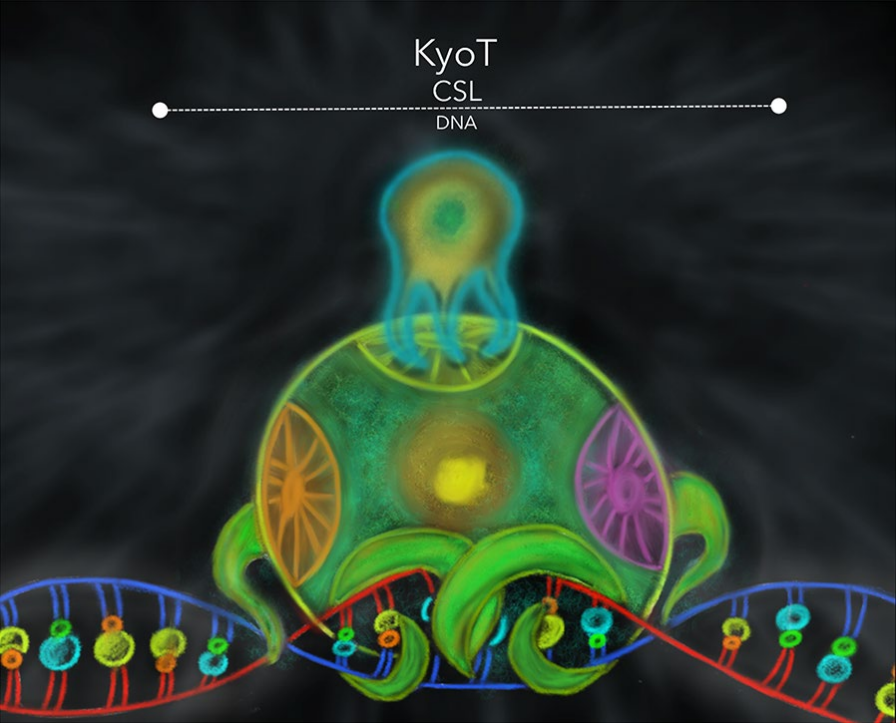
KyoT-CSL. The interaction of KyoT 1 and 3 proteins with the transcription factor is mapped at the central region of CSL. This interaction still proposed the exclusive and antagonistic competition between the co-activators and the co-repressor in the NSP. Concentration of the KyoT proteins seems to play also an important role in the regulation control of genes expression, which are commune characteristics of the repression complexes in this signaling context

KyoT 2 (containing LIM1 and 2 domains) and KyoT 3 (containing LIM1, 2 and 3 domains) proteins, but not KyoT 1 protein, possess the singularity to interact with RBPJ-κ protein [93, 96]. Interaction occurs at the central region of RBPJ-κ, the BTD (Fig. 6). The DNA-bound activator complex of NSP has been solved in great detail: the NTD and the BTD of CSL make the DNA contacts, whereas the BTD and the CTD bind to NICD and Mam. Thus the BTD is critical for activator and repressor complex formation alike. The interaction between KyoT/RBPJ-κ and NICD/RBPJ-κ are exclusive and antagonistic [93, 95, 96]. This way, KyoT 2 has the capacity to antagonize the gene expression mediated by NICD in a concentration-dependent manner. In other words, when the KyoT 2 concentration increases inside the cell nucleus, it can provoke a sequential gene inactivation of Notch dependent genes, because the KyoT 2 competes with NICD for the binding of RBPJ-κ [93, 96]. KyoT 3 likewise binds RBPJ-κ and inhibits *Hes*-*1* gene activation, a prime Notch target gene which encodes a bHLH transcriptional repressor [94]. These data together could mean that both proteins, KyoT 2 and KyoT 3, truly work by antagonizing gene expression under NSP control, but in a different temporality that gives as result a different grade of cellular specialization.

The competition between KyoT 2 and NICD for the binding of the RBPJ-κ BTD is quite different from the interaction between Su(H) and H. It has been demonstrated that H binds only to the CTD of Su(H), and there to sites different from NICD [17]. This means that these two proteins, KyoT 2 and KyoT 3 conserved the ability to regulate NSP genes in specialized tissues during different steps of embryo formation, but in a different way as H does [93, 95, 96].

### RITA protein

**Fig. 7:**
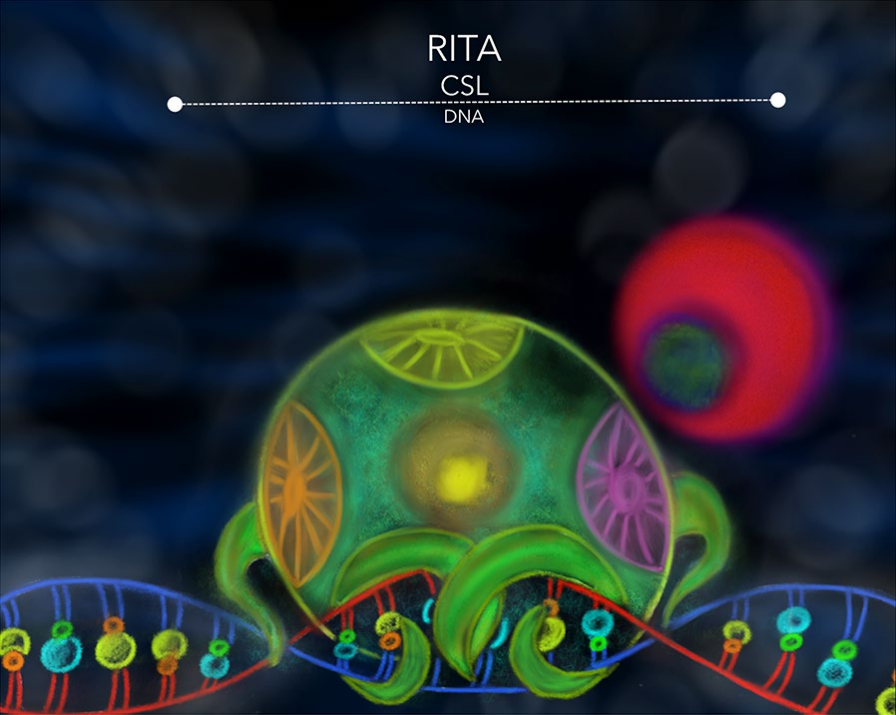
RITA-CSL. This interaction represents a different, but still important mechanism of regulation of the NSP. In this case chromatin modification is not involved, instead the complex is shuttle out of the nucleus by activity of RITA. The CSL interacting domain with RITA is mapped at the CTD

The *R*BPJ-κ *i*nteracting and *t*ubulin *a*ssociated protein (RITA) was identify and characterized by Wacker et al. [98]. The 36 kDa RITA protein contains a tubulin interaction domain, a functional nuclear localization signal (NLS), a nuclear export signal (NES), and the RBP-J-interaction domain at the central region of the protein. These domains are used by RITA to interact with nuclear RBPJ-κ protein and to shuttle the transcription factor out of the nucleus, negatively regulating the Notch signaling pathway in vertebrates such as *Xenopus laevis* and the mouse [98]. Brockmann et al. [99] analyzed the Su(H) interaction capacity and effects of ectopically expressed RITA in *Drosophila*, where no RITA-homologue has been detected in the genome. In those experiments they demonstrated that RITA interacts with the C-terminal domain of the Su(H) (Fig. 7) in vitro and in vivo, even though overexpression of RITA in *Drosophila* had not significant phenotypic effects [99].

## Conclusions

What do we know so far? In the NSP context, the transcriptional complexes involving CSL protein members have the capacity to modify the chromatin structure to allow accurate regulation of gene expression, depending on the proteins that are assembling the transcriptional complex. This generalization is possible due to the fact that the basic core of the highly conserved NSP has only a small number of reiteratively used proteins [4, 7]. In addition, this process is dependent on the cellular context. These core proteins are capable to activate/inactivate different target genes, thereby controlling cellular specification during embryo development, according to the ON–OFF (Switch) model [9, 79]. This model may be oversimplified, because we have to keep in mind that embryo development means a massive diversity of developmental processes occurring practically at the same time. For this reason, a NSP dysfunction is implicated in many disorders, even the death of the embryo. Lately it has become apparent, that the negative gene regulation of NSP is highly relevant for its pleiotropic effects. Evidently the cellular context (cell position, differentiation state and nuclear topology) is an important element to obtain the correct gene expression under the NSP control. All involved elements define which proteins should be part of the transcriptional complexes. Taken together, these components define gene regulation.

As we can see, all of the proteins stated above share the capability of assembling complexes with the CSL protein family. All of them are specialized in antagonizing gene expression engaging NSP at different cellular contexts [8, 25, 31, 32, 50, 83, 86, 93]. This ensures a correct cell specification during embryo development. Despite all these proteins can associate with the CSL proteins, H associates at a different domain than the known antagonists in vertebrates [17, 36, 80]. One exception is the SHARP/MINT protein, which interacts in addition to BTD also at the CTD domains [82]. As referred before, SHARP interaction with CtIP/CtBP complex may suggest an HDAC-independent repression, as demonstrated by Koipally and Georgopoulos in 2002 [89]. This property gives those proteins an extraordinary versatility to work as transcriptional elements in different cellular backgrounds under the control of different signaling pathways.

Another kind of negative regulation is represented by RITA [98, 99]. RITA antagonizes NSP by depleting the CSL transcription factors from the nucleus [98, 99]. Unlike most other transcription factors, CSL depends piggyback import by either NICD or its corepressors [29, 40, 78]. This shuttle activity was described previously by Maier et al. in 1999 [78], when they addressed the nuclear localization of H protein in *Drosophila* and observed that cells devoid of H protein contained less of nuclear CSL/Su(H) protein, whereas those with ectopic H protein caused a strong nuclear accumulation of Su(H) [78]. In the meantime it was shown that the CTD of Su(H) is sufficient for nuclear import by H protein [78, 100].

Despite the excellent characterization of H protein as major NSP antagonist in flies, H has been identified until now only in insects. Since it is necessary to regulate the correct gene expression at any cellular context during the development of any organism, the fine modulation mechanisms of NSP has been adapted in the various lineages to account for the respective specific requirements [99]. Noteworthy, Maier et al. [17] recently demonstrated that H protein is capable to bind the mouse CSL-CBF1 transcription factor, indicating that the binding site of H protein within the CTD domain of CSL proteins is well conserved. This raises the possibility for proteins containing a conserved H-type CSL-binding domain also in vertebrates. Such proteins may act as transcriptional coregulators using the H-binding site on CTD, which may be indicative of an as-yet-unidentified mode of NSP repression in mammals [17, 22].

## Abbreviations

BEN: *B*ANP, *E*5R and *N*AC1 proteins
BTD: *b*eta-*t*refoil *d*omain
bHLH: *b*asic *h*elix-*l*oop-*h*elix protein family
CID: *C*SL *i*nteracting *d*omain
CIR: *C*BF-1 *i*nteracting *r*epressor
CSL: *C*BF-1, *S*uppressor of Hairless, *L*ag-1 proteins family
CtBP: *C*-*t*erminal *b*inding *p*rotein
CTD: *C*-*t*erminal *d*omain
DSL: *D*elta/*S*errate, *L*AG-2, Dll-3 and 4/Jagged proteins family
Dll-1: Delta 1
E(spl): *E*nhancer of *spl*it protein
FGF: *f*ibroblast *g*rowth *f*actor signaling pathway
Gro: *Gro*ucho protein
H: *H*airless protein
HAT: *h*istone *a*cetyl *t*ransferase
HDAC: *h*istone *d*e*ac*etylase
Hh: *H*edge*h*og signaling pathway
ICM: *i*nternal *c*ell *m*ass
LIM: *L*in11, *I*sl-1 and *M*ec-3 proteins
Lnfg: *l*u*n*atic *f*rin*g*e
MAP: *m*itogen-*a*ctivated *p*rotein
MINT: *M*sx2 *i*nteracting *n*uclear *t*arget
MOG-3: *m*asculinization *o*f the *g*erm line
NCoR: *n*uclear *co*-*r*epressor
NES: *n*uclear *e*xport *s*ignal
NICD: *N*otch *i*ntracellular *d*omain
NSP: *N*otch *s*ignaling *p*athway
NTD: *N*-*t*erminal *d*omain
NuRD: *n*uclear *r*emodeling *d*eacetylase
PSM: *p*re*s*omitic *m*esoderm
RRM: *R*NA *r*ecognition *m*otifs
RTK/P: *r*eceptor *t*yrosine *k*inase/*p*hosphatase signaling pathway
SHARP: *S*MRT/*H*DAC-1 *a*ssociated *r*epressor *p*rotein
SMRT: *s*ilencing *m*ediator *r*etinoid and *t*hyroid hormone
SOP: *s*ensory *o*rgan *p*recursor cell
SPEN: *sp*lit *en*ds
SPOC: *S*pen *p*aralog and *o*rtholog *C*-terminal domain
Su(H): *Su*ppressor of *H*airless protein
Tbx: transcription factor family proteins
TE: *t*rophectoderm *e*pithelium
Wnt: wingless signaling pathway
